# MRI with generalized diffusion encoding reveals damaged white matter in patients previously hospitalized for COVID-19 and with persisting symptoms at follow-up

**DOI:** 10.1093/braincomms/fcad284

**Published:** 2023-10-22

**Authors:** Deneb Boito, Anders Eklund, Anders Tisell, Richard Levi, Evren Özarslan, Ida Blystad

**Affiliations:** Department of Biomedical Engineering, Linköping University, S-58183 Linköping, Sweden; Centre for Medical Image Science and Visualization (CMIV), Linköping University, S-58183 Linköping, Sweden; Department of Biomedical Engineering, Linköping University, S-58183 Linköping, Sweden; Centre for Medical Image Science and Visualization (CMIV), Linköping University, S-58183 Linköping, Sweden; Division of Statistics and Machine learning, Department of Computer and Information Science, Linköping University, S-58183 Linköping, Sweden; Centre for Medical Image Science and Visualization (CMIV), Linköping University, S-58183 Linköping, Sweden; Department of Radiation Physics, Linköping University, S-58185 Linköping, Sweden; Department of Health, Medicine and Caring Sciences, Linköping University, S58183 Linköping, Sweden; Centre for Medical Image Science and Visualization (CMIV), Linköping University, S-58183 Linköping, Sweden; Department of Health, Medicine and Caring Sciences, Linköping University, S58183 Linköping, Sweden; Department of Rehabilitation Medicine in Linköping, Linköping University, S-58185 Linköping, Sweden; Department of Biomedical Engineering, Linköping University, S-58183 Linköping, Sweden; Centre for Medical Image Science and Visualization (CMIV), Linköping University, S-58183 Linköping, Sweden; Centre for Medical Image Science and Visualization (CMIV), Linköping University, S-58183 Linköping, Sweden; Department of Health, Medicine and Caring Sciences, Linköping University, S58183 Linköping, Sweden; Department of Radiology in Linköping, Linköping University, S-58185 Linköping, Sweden

**Keywords:** MRI, Q-space trajectory imaging, microscopic fractional anisotropy, fractional anisotropy, COVID-19

## Abstract

There is mounting evidence of the long-term effects of COVID-19 on the central nervous system, with patients experiencing diverse symptoms, often suggesting brain involvement. Conventional brain MRI of these patients shows unspecific patterns, with no clear connection of the symptomatology to brain tissue abnormalities, whereas diffusion tensor studies and volumetric analyses detect measurable changes in the brain after COVID-19. Diffusion MRI exploits the random motion of water molecules to achieve unique sensitivity to structures at the microscopic level, and new sequences employing generalized diffusion encoding provide structural information which are sensitive to intravoxel features. In this observational study, a total of 32 persons were investigated: 16 patients previously hospitalized for COVID-19 with persisting symptoms of post-COVID condition (mean age 60 years: range 41–79, all male) at 7-month follow-up and 16 matched controls, not previously hospitalized for COVID-19, with no post-COVID symptoms (mean age 58 years, range 46–69, 11 males). Standard MRI and generalized diffusion encoding MRI were employed to examine the brain white matter of the subjects. To detect possible group differences, several tissue microstructure descriptors obtainable with the employed diffusion sequence, the fractional anisotropy, mean diffusivity, axial diffusivity, radial diffusivity, microscopic anisotropy, orientational coherence (*C*_c_) and variance in compartment’s size (*C*_MD_) were analysed using the tract-based spatial statistics framework. The tract-based spatial statistics analysis showed widespread statistically significant differences (*P* < 0.05, corrected for multiple comparisons using the familywise error rate) in all the considered metrics in the white matter of the patients compared to the controls. Fractional anisotropy, microscopic anisotropy and *C*_c_ were lower in the patient group, while axial diffusivity, radial diffusivity, mean diffusivity and *C*_MD_ were higher. Significant changes in fractional anisotropy, microscopic anisotropy and *C*_MD_ affected approximately half of the analysed white matter voxels located across all brain lobes, while changes in *C*_c_ were mainly found in the occipital parts of the brain. Given the predominant alteration in microscopic anisotropy compared to *C*_c_, the observed changes in diffusion anisotropy are mostly due to loss of local anisotropy, possibly connected to axonal damage, rather than white matter fibre coherence disruption. The increase in radial diffusivity is indicative of demyelination, while the changes in mean diffusivity and *C*_MD_ are compatible with vasogenic oedema. In summary, these widespread alterations of white matter microstructure are indicative of vasogenic oedema, demyelination and axonal damage. These changes might be a contributing factor to the diversity of central nervous system symptoms that many patients experience after COVID-19.

## Teaser

Boito *et al*. investigated the integrity of brain white matter microstructure of patients previously hospitalized for COVID-19 and suffering from persisting symptoms at follow-up. Advanced diffusion MRI acquisition and analysis revealed widespread microstructural alterations of the white matter, compatible with vasogenic oedema, demyelination and axonal damage.

## Graphical Abstract

**Graphical Abstract:**
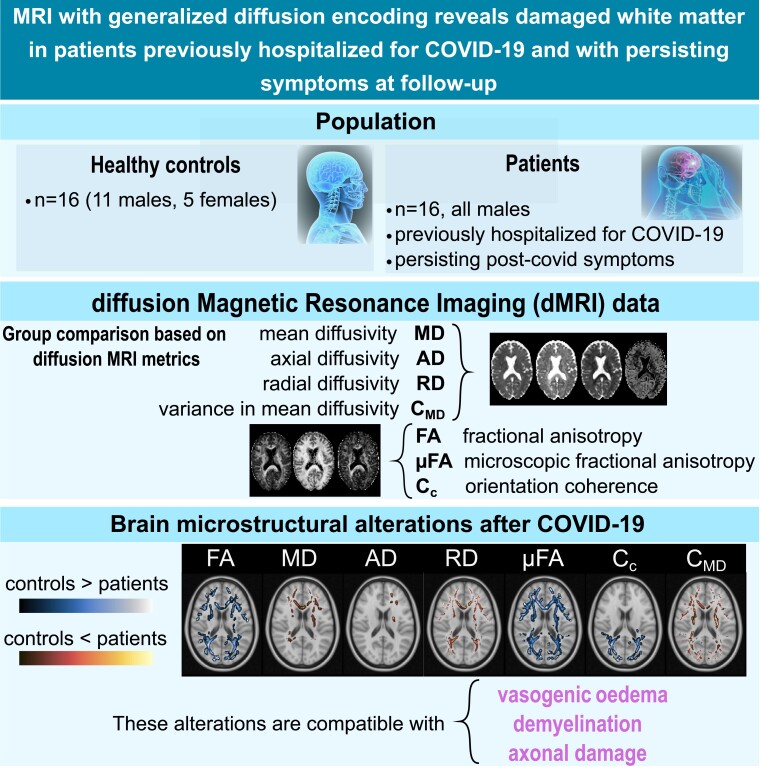

## Introduction

### COVID-19 and the brain

Since the first wave of the COVID-19 pandemic, it has become clear that the infection caused by the corona virus may go with long-term sequelae, affecting a considerable percentage of the population on a long-term basis (5%,^1^ 6.5–28.5%^2^). Symptoms are diverse and often suggest brain involvement, manifesting in problems such as fatigue, cognitive impairment, depression and anxiety.^3^ Persisting problems can linger for several years, affecting patients irrespective of the initial disease severity.^4^ Brain findings on MRI in the acute phase of the infection during the first pandemic were often associated with vascular changes, such as ischaemic and haemorrhagic events.^5,6^ At later follow-up, conventional clinical MRI shows unspecific patterns of structural changes,^7,8^ whereas diffusion tensor studies and volumetric analyses detect measurable changes in the brain after COVID-19.^9-11^ In this work, advanced diffusion MRI (dMRI) is used to quantitatively analyse the properties of brain tissue in 16 patients previously hospitalized for COVID-19 and with persisting symptoms after the infection at 7-month follow-up. The analysis is based on a comparison between this group and 16 controls with no post-COVID symptoms and who were not hospitalized for COVID-19.

### dMRI and Q-space trajectory imaging

dMRI is an imaging technique with extreme sensitivity to tissue structure on the micrometre level. The structural properties of the tissue can be retrieved by measuring, typically with a pair of pulsed magnetic field gradients,^12^ and modelling the random motion of water molecules.^13-15^ Barriers in the tissue guide the diffusion process, thus imprinting their presence and characteristics into the measurement. Models can then be applied to the measured diffusion signal to retrieve these features.

In neurological medical practice, dMRI has become a staple for fast detection of acute brain ischaemia and for studying brain connectivity.^16^ Diffusion MR images obtainable with standard clinical MR systems are however typically limited to 2–3 mm spatial resolution while being sensitive to structures at the microscopic level. This means that all the contributions from different micrometre-level features within a voxel conflate and are typically lost when only voxel-scale metrics, such as those obtainable with diffusion tensor imaging (DTI),^17^ are used.

Recent efforts directed towards resolving this limitation resulted in innovative dMRI encodings and methods. While standard dMRI acquisitions rely on single diffusion encoding (SDE), i.e. diffusion being measured along a single direction at a time, new protocols involving simultaneous diffusion measurements in multiple directions have been developed.^18-23^ This has allowed for retrieval of more specific features of the tissue microstructure which prompted interest towards bringing these methods into the clinic.

**Figure 1:**
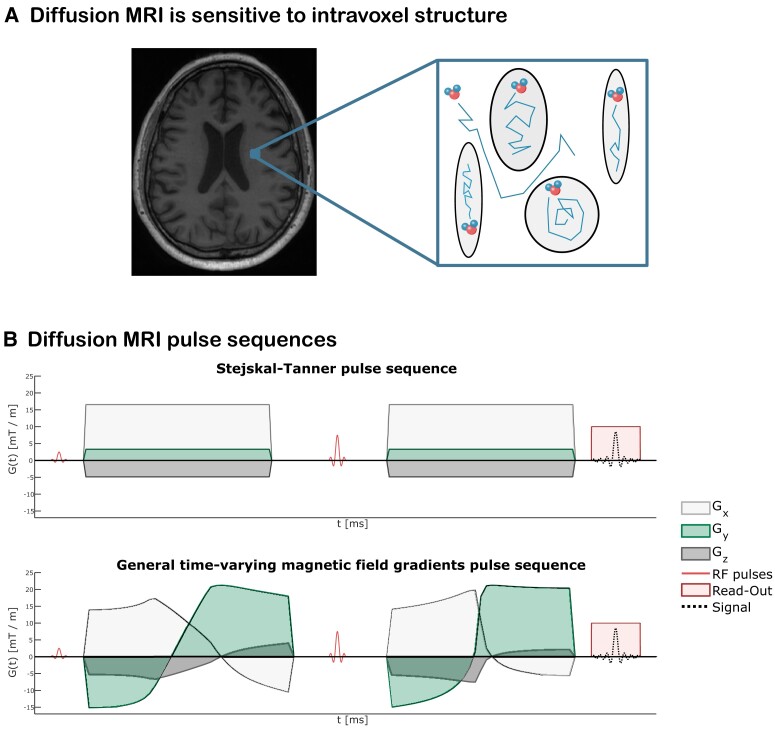
**dMRI pulse sequences and sensitivity to intra-voxel features.** (**A**) dMRI images achieve millimetre-scale spatial resolution while probing structures at the microscopic level, meaning that intra-voxel microstructural features are available. The analysis method employed here pictures the intra-voxel environment as being composed of several non-exchanging pores, each modelled with a diffusion tensor. (**B**) dMRI pulse sequences. *Top* row: conventional Stejskal–Tanner pulse sequence employing trapezoidal magnetic field gradients before and after the refocussing radiofrequency (RF) pulse to achieve diffusion sensitization. *Bottom* row: a pulse sequence utilizing time-varying magnetic field gradients for diffusion encoding. In both experiments, the relative intensities of the *x*, *y* and *z* components of the gradients determine the gradient direction at a given time.

Q-space trajectory imaging (QTI)^24^ is a diffusion imaging technique that utilizes diffusion data acquired with general time-varying magnetic field gradients,^25,26^ which allow time-efficient measurements of water displacement in multiple directions. The diffusion waveforms employed in QTI are typically referred to as linear tensor encoding (LTE), planar tensor encoding (PTE) and spherical tensor encoding (STE), for 1D, 2D and 3D diffusion measurements, respectively. For analysing the collected data, QTI employs a multicompartment model for the tissue microstructure, where each voxel is envisioned as being composed of many diffusion tensors.^27^ In a sense, this method alleviates the limitation of DTI where all intravoxel microscopic tensors are condensed into one, which represents their average. Figure 1A shows this idea of modelling each voxel as a collection of separate environments where water is freely diffusing within and in between them, exploiting dMRI’s sensitivity to finer-than-voxel size features. Figure 1B shows two diffusion-weighted pulse sequences, one where conventional trapezoidal gradients are used to achieve diffusion sensitization and one where general time-varying magnetic field gradients are employed to achieve diffusion sensitization.

**Figure 2:**
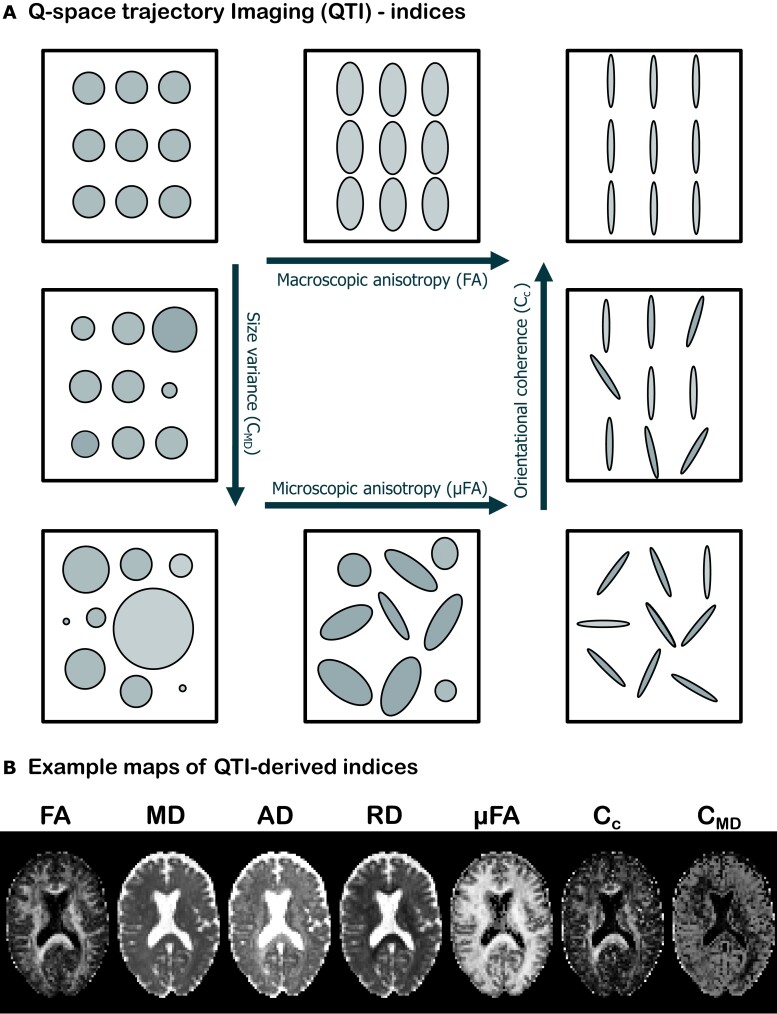
**QTI indices.** (**A**) Indices of tissue microstructure features retrieved with QTI. The arrows indicate the direction of increasing value for each metric [macroscopic anisotropy, quantified by FA, µFA, size variance (*C*_MD_) and orientational coherence (C_c_)]. All metrics are bounded between 0 and 1. (**B**) Example maps of QTI-derived indices for one of the healthy volunteers included in the study.

The QTI analysis provides several quantities akin to various ‘stains’ used in histology, which capture different aspects of the tissue microstructure. Figure 2A shows pictorially four such measures and how they change based on the characteristics of the intravoxel environment. Macroscopic anisotropy is typically quantified by fractional anisotropy (FA),^28^ which measures the voxel-level degree of diffusion anisotropy and orientational coherence. FA is 0 when voxel-averaged diffusion is equally probable in all directions and 1 when diffusion occurs in only one direction. In terms of the multicompartment picture, this means that in order for FA to take the value 1, diffusion in all microenvironments needs to share the same preferred orientation. In the case where all microenvironments exhibit anisotropic diffusion but not along the same direction, FA will take a value lower than 1. Microscopic anisotropy (µFA) captures the degree of anisotropy on a local level (i.e. for each microscopic environment), and it is thus insensitive to the relative orientation of the compartments. This metric then takes the value 0 when diffusion is isotropic in all microenvironments, while its value is close to 1 when the voxel comprises highly anisotropic microenvironments, irrespective of whether they share a common preferred orientation. The degree to which diffusion exhibits a global preferred orientation is captured by the orientational coherence (*C*_c_) parameter. When diffusion is locally anisotropic but not globally, this parameter takes the value 0. When diffusion is instead both locally and globally anisotropic, its value is close to 1. The size variance (*C*_MD_) parameter reflects the degree of variation in compartment size within each voxel. If compartments are all the same size, this parameter takes the value 0, whereas when compartments have different sizes, it takes a value closer to 1. Figure 2B shows an example of how these parameters look for one of the healthy subjects enrolled in this study.

Recent studies investigated the sensitivity of QTI-accessible metrics, like μFA and *C*_MD_, on various cerebral diseases such as schizophrenia,^24^ brain tumours,^29^ epilepsy,^30^ multiple sclerosis^31^ and Parkinson’s disease.^32^ All these studies showed the potential for these metrics to characterize the disease more specifically, when compared to quantities obtainable with conventional dMRI methods such as DTI.

### Aim

The aim of this study was to compare the brain white matter of patients previously hospitalized for COVID-19 and with subsequent persisting symptoms after the infection, with a matched healthy control group, using advanced dMRI. We hypothesized that the symptomatology reported by the patients at 7-month follow-up would be reflected by altered water diffusion patterns, due to microstructural changes in the brain tissue.

Most previous dMRI studies on brain-related effects of COVID-19 employed conventional dMRI quantities and methods (apparent diffusion coefficient^33^ and DTI^9,34,35^). In this work, the dMRI data were instead acquired using general time-varying magnetic field gradients and analysed using QTI. A second aim was therefore to assess the sensitivity of the diffusion metrics accessible via QTI to possible microstructural alterations and to determine whether the newly available quantities add relevant information when compared to DTI-derived metrics.

## Materials and methods

### Participants

Sixteen patients from the Linköping COVID-19 Study cohort^7,36^ previously hospitalized with a laboratory-confirmed (polymerase chain reaction) COVID-19 diagnosis during the first wave of the pandemic in the spring of 2020, and with persisting symptoms at the clinical evaluation during follow-up, were included for an extended MR examination. Premorbid level of function was assessed, and individuals with severe frailty and severe pre-existing comorbidities were not included.^7^ Mean patient age was 60 years (41–79 years), and all were men. Eleven of these patients had been in ventilator care for a mean of 15 days (7–41 days). The MR scan was performed after outpatient follow-up on average 230 days after the admission to the hospital (204–256 days). This is a subgroup of the cohort reported in Hellgren *et al*.^7^ An age-matched control group of 16 healthy individuals with no neurological disease, no symptoms of post-COVID condition and not previously hospitalized for COVID-19 was recruited [mean age 58 years (46–69 years), 11 men and 5 women]. The control group did not undergo infection status assessment (see Table 1 for details on demographics). Data were collected with ethical approval from the Swedish ethical review authority Dnr 2020-03029, 2015/13-31, and informed written consent was obtained from all participants.

**Table 1:** Description of participants

Participants	Patients	Healthy controls
Gender M/F	16/0	11/5
Age	60 (41–79 years)	58 (46–69 years)
Ventilator care	11	N/A
Time in ventilator	15 (7–41 days)	N/A
Time to follow-up	230 (204–256 days)	N/A
Fazekas 0	4	6
Fazekas 1	8	10
Fazekas 2	4	

Time is given in mean with range. Time to follow-up is from hospital admission. N/A, not applicable.

### Neuroimaging data acquisition

The 32 subjects were scanned with a 48-channel head coil on a clinical GE Signa Architect 3T MR scanner at Linköping University Hospital, using the clinical protocol described in Hellgren *et al.*,^7^ with the addition of the advanced diffusion sequence. Briefly, the clinical protocol included axial T_2_–fluid-attenuated inversion recovery, axial T_2_–fast spin echo (T_2_–FSE), T_1_–FSE, 3D T_1_–gradient echo, axial diffusion weighted imaging and axial susceptibility-weighted imaging.

The diffusion MR images were collected using a QTI protocol comprising 122 measurements of which 6, 6, 16 and 30 LTE measurements at *b* = 100, 700, 1400 and 2000 s/mm^2^; 6, 10 and 15 PTE measurements at *b* = 100, 1000 and 2000 s/mm^2^; 6, 6, 10 and 10 STE measurements at *b* = 100, 700, 1400 and 2000 s/mm^2^; and 1 measurement without diffusion weighting. The imaging parameters were echo time = 122 ms, repetition time = 3289 ms, field of view 240 × 240 × 156 mm^3^, matrix size = 80 × 80 × 39 and voxel size = 3 × 3 × 4 mm^3^. Total QTI scan time was 6 and 45 s.

### Data analyses

#### Subject-level preprocessing

After converting the collected DICOM images to NIFTI using ‘dcm2niix’,^37^ the data were preprocessed for head motion and eddy current correction using the ‘eddy’ tool from ‘FSL’^38^ interfaced via ‘Mrtrix3’.^39^ Susceptibility distortion correction was not applied, since the diffusion data were only collected with a single phase-encoding direction. The QTI maps were then obtained using the QTI ± framework,^40^ which achieves robust estimates throughout the brain by enforcing a set of mathematical constraints.^40,41^

#### Group comparisons

To detect possible group differences in terms of the microstructure metrics obtainable with QTI, the FA, µFA, C_c_, mean diffusivity (MD), axial diffusivity (Ad), radial diffusivity (RD) and C_MD_ maps were analysed using the tract-based spatial statistics (TBSS)^42^ framework. Briefly, the FA maps of all subjects were non-linearly co-registered using the FMRIB58_FA template image as target and subsequently affine aligned to the MNI152 space. The FA maps were then averaged, and the average FA map was used to derive a skeleton of voxels which should represent the white matter tracts common to all subjects. A threshold of 0.2 (selected according to the TBSS user recommendations and after visual inspection) was used to refine the mean FA skeleton. Each subject’s FA map was then projected onto the skeleton prior to performing the statistical analysis. One-sided two-sample unpaired *t*-tests were employed to detect FA differences between the two groups, testing both directions (FA^controls^ > FA^patients^ and FA^controls^ < FA^patients^). This was performed using the ‘randomise’ function in FSL,^43^ as recommended by the TBSS user guide. Five thousand permutations were used to build the null hypothesis, and the threshold-free cluster enhancement option was used as it often results in a higher statistical power compared to cluster extent thresholding.^44^ Age and sex were included as covariates, to make sure that any group difference is not due to age or sex. The same procedure was then repeated for the other QTI-derived maps using the registration matrices and projection vectors obtained from the FA analysis to co-register the images and populate the skeleton, respectively.

## Results

As reported in Hellgren *et al*.,^7^ the findings of the conventional images in the patient cohort were unspecific with white matter lesions and some abnormalities on susceptibility-weighted images. There were no clinically significant findings of the conventional images of the control group, only unspecific white matter lesions. The Fazekas scores are presented in Table 1.

### TBSS results

**Figure 3:**
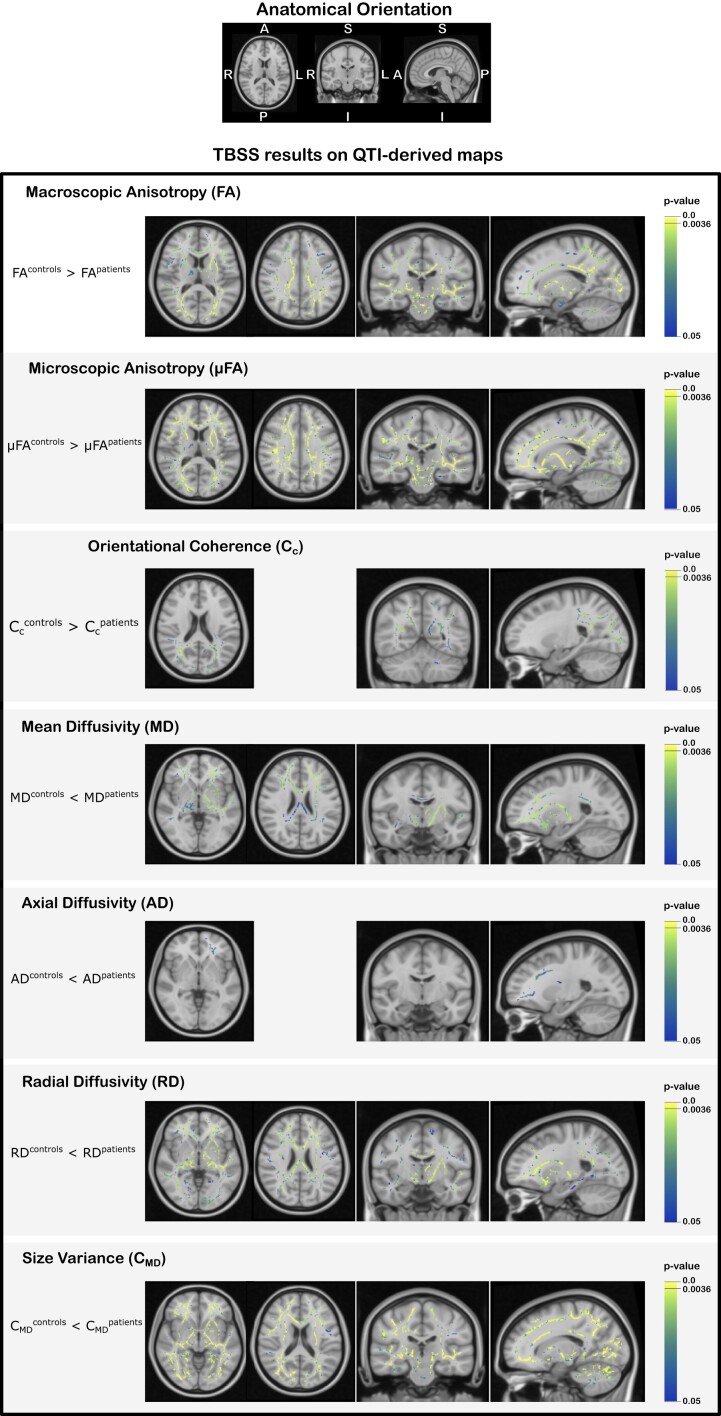
**TBSS results on QTI-derived maps.** TBSS results for the QTI-derived maps. Each row shows the results of the comparison between the mean value of each map for the two groups, with symbols ‘>’ and ‘<’ indicating the direction of the test. The voxels coloured in the blue–yellow colour scale depict the locations on the skeleton where statistically significant differences emerged (*P* < 0.05, FWE corrected). The *P* < 0.0036 represents the statistical threshold for significance when correcting the familywise error for multiple contrasts.

**Figure 4:**
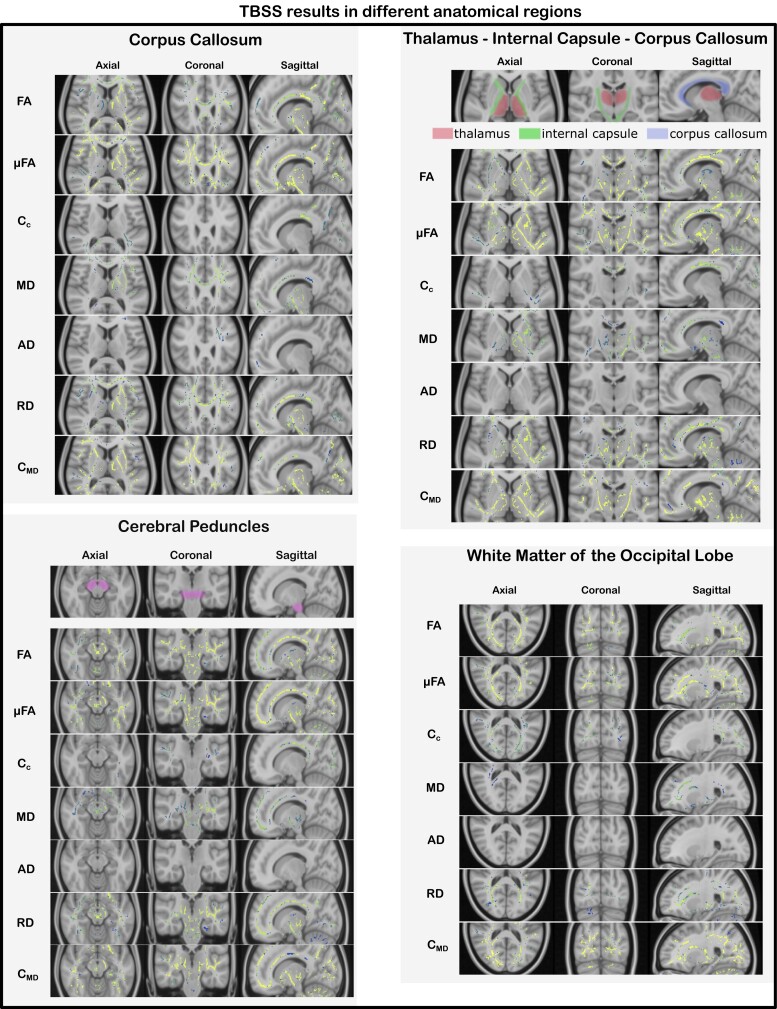
**TBSS results in different anatomical areas.** TBSS results for all the considered QTI-derived metrics in different brain regions and anatomical structures for the tests which resulted in statistically significant differences (*P* < 0.05, FWE corrected) between the two groups (FA^controls^ > FA^patients^, µFA^controls^ > µFA^patients^, *C*_c_^controls^ > *C*_c_^patients^, MD^controls^ < MD^patients^, AD^controls^ < AD^patients^, RD^controls^ < RD^patients^ and *C*_MD_^controls^ < *C*_MD_^patients^). Changes were widespread, affecting all lobes of the brain.

The TBSS analysis showed widespread differences in all QTI-derived metrics in the white matter of the patients compared to the controls. Figure 3 shows exemplary results from the statistical analysis performed within the TBSS framework in different slices for a statistical significance level of *P* < 0.05, corrected for multiple comparisons using familywise error rate (FWE). Figure 4 shows the TBSS results in different anatomical regions for *P* < 0.05, FWE-corrected for multiple comparisons. In these figures, the colour map is chosen to highlight results for *P* < 0.0036, where a Bonferroni correction for testing over multiple contrasts (seven microstructure metrics tested for the mean of each group being either greater or lower than the other group’s mean, leading to *P* = 0.05/14 = 0.0036) is applied.^45^ The results of the TBSS analysis can also be explored in NeuroVault (https://identifiers.org/neurovault.collection:13799) where we share all the statistical maps.

In general, the two measures of diffusion anisotropy, FA and µFA, were found to be lower in the patients’ white matter, while the MD and the variance in compartment size *C*_MD_ were found to be higher in the patient group. When looking at the axial and radial diffusivities (AD and RD), RD had higher values in the whole brain in the patient group, whereas differences in AD, with higher values for patients, were limited to the white matter of the left frontal lobe. The parameter describing the structural orientational coherence within the voxel, *C*_c_, was found to be lower for the patient group.

**Figure 5:**
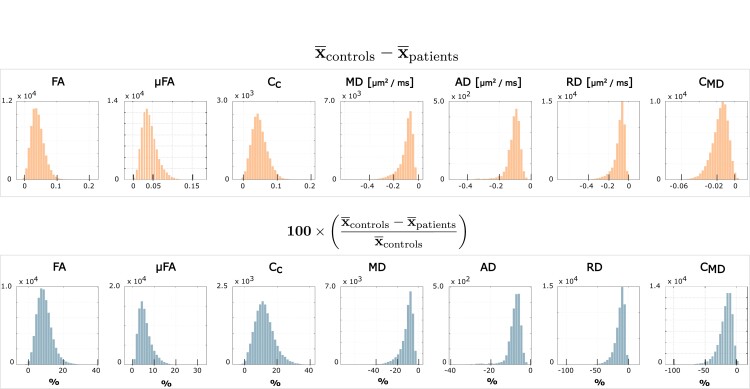
**Quantitative QTI metric differences between the control and patient groups.** Absolute (first row) and percentage (second row) differences between the group-averaged QTI-derived metrics for the voxels lying on the skeleton obtained within the TBSS analysis. Only the voxels presenting statistically significant differences (*P* < 0.05, FWE corrected) were included. FA, µFA, *C*_c_ and *C*_MD_ take values in the range [0, 1], while MD, AD and RD take value in the range [0, 3] µm^2^/ms. The symbol in the *y*-axis label indicates that for each of the metrics, we consider the group-averaged value.

**Figure 6:**
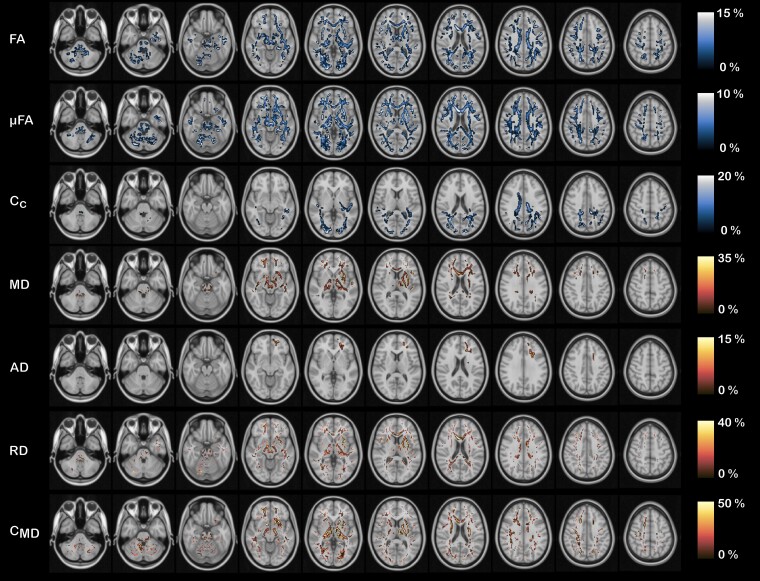
**Visualization of the QTI metric differences in percentage across the brain.** Visualization of the QTI metric differences in percentage between patients and controls across the brain. FA, µFA, RD and *C*_MD_ present widespread differences. Changes in MD and AD are localized in the frontal part of the brain, while changes in *C*_c_ are localized in the dorsal part of the brain. The cold black–blue–white colours indicate a reduction in the metric, while the warm black–red–yellow colours indicate an increase in the metric. Only the voxels presenting statistically significant differences (*P* < 0.05, FWE corrected) were included.

Quantification of the inter-group difference for each QTI-derived metric over the skeleton voxels exhibiting statistical significance is presented in Fig. 5. The histograms show the distributions of differences between the metric’s mean (indicated with $\bar{x}$) for the two groups. A positive value indicates that the mean for the healthy controls is higher compared to the patients, and vice versa. The results in percentage units show that all the metrics present quite pronounced differences (median values 8.5, 5.2, 11.7, −9.2, −7.3, −11.9 and −15.3%, for FA, µFA, *C*_c_, MD, AD, RD and *C*_MD_, respectively). Figure 6 shows where these differences are localized in the brain. FA, µFA, C_MD_ and RD presented the most widespread differences between the two groups, showing statistically significant differences (*P* < 0.05, FWE corrected) in, respectively, 44, 56, 47 and 36% of the analysed white matter voxels. MD, AD and *C*_c_ presented differences in, respectively, 17, 1 and 12% of the skeleton voxels. Changes in MD and AD were mostly localized in the frontal part of the brain, while differences in *C*_c_ were found occipitally.

## Discussion

In this study, we found widespread changes in the white matter of the brain in patients previously hospitalized for COVID-19 with persisting symptoms at follow-up, compared to a matched healthy control group, as revealed by advanced dMRI. As seen in other neuroinflammatory and neurodegenerative conditions,^46,47^ patients show a decrease in anisotropy-related measurements and an increase in diffusivity-related metrics compared to the controls, indicating a general loss of tissue integrity at the microstructural level as well as diffuse oedema. As shown in the histograms in Fig. 5, the amount by which the metrics differ between the two groups can be quite pronounced. Displaying these differences in multiple axial slices highlights how some metrics (FA, µFA, RD and *C*_MD_) are widely affected, while others (MD, AD and *C*_c_) exhibit more localized changes in the frontal and parietal lobes.

Focusing on voxel-level metrics (FA, MD, RD, AD), these changes were seemingly due to an increase in RD, while the AD was essentially unaltered. Other publications^33-35^ where dMRI was also employed to study COVID-19-induced alterations in the brain report similar trends. Though not specific to any biological tissue feature, these metrics have been correlated with different neuronal damaging processes. Increased values of RD were found to be related to demyelination, while alterations of AD were representative of axonal damage.^48^ Our results would then suggest an underlying process of myelin damage, which is reasonable given that demyelination is an unspecific reaction when damage occurs to the white matter. Demyelination can be a product of inflammatory or infectious processes in the CNS and has been reported in association with COVID-19.^49,50^

Rau *et al.* employed the diffusion microstructure imaging methods for a three-compartment biophysical model [similar in spirit to the neurite orientation dispersion and density imaging (NODDI)^51^ model] for interpreting the dMRI data of COVID-19 patients with neurological symptoms.^52^ In their work, a volume fraction shift from the intra- and extra-axonal spaces to the free water compartment was reported and interpreted as being representative of vasogenic oedema. The reduction in FA and increase in MD and RD (as well as increase in *C*_MD_, discussed later) found in our study could also fit with this explanation. However, it is important to stress that conventional dMRI data (as the one used in Rau *et al.*^52^) has been shown to not contain enough information to allow for reliable estimation of such biophysical model parameters^53,54^; thus, care should be taken when interpreting the results. Moreover, due to the unspecific nature of metrics such as FA and MD with respect to biological features, drawing conclusions on the exact physiological mechanisms underpinning these changes is problematic.

The major advantage deriving from combining the advanced diffusion acquisition sequence and analysis employed in this study is that it allows estimation of intra-voxel microstructural features, thus complementing the voxel-level information obtainable with standard dMRI protocols and methods. This is particularly relevant when competing intra-voxel effects lead to opposite directional changes in voxel-level metrics,^54-56^ thus limiting their sensitivity and interpretability. Conversely, separating each effect’s contribution has already been shown to boost specificity when characterizing different brain diseases, such as brain tumours^29^ and multiple sclerosis.^31^

Similarly to what was reported in those studies, the results obtained here suggest µFA as a more sensitive (and specific) metric for detecting microstructural changes compared to FA. Conversely to FA, µFA is a measure of diffusion anisotropy unconfounded by orientation dispersion, this latter being quantified by *C*_c_. Therefore, having access to these two metrics (µFA and *C*_c_) not only allows for increased specificity to the exact mechanism underpinning changes in diffusion patterns but may also highlight alterations in the microstructure which would not appear when FA alone is considered. Looking at the results in Figs 3–5, one can postulate that, given the predominant alterations in µFA compared to *C*_c_, the observed change in diffusion anisotropy is mostly due to loss of local anisotropy rather than white matter fibre coherence disruption. Since µFA has been proposed as a measure reflecting axons rather than myelin,^57,58^ this suggests widespread axonal damage resulting from the severe COVID-19 infection. Therefore, considering the observed results on voxel and intra-voxel metrics, alterations in the microstructure seems to be due to damage to both myelin and axons. We stress that this argument would not have been possible if only the voxel-level metrics, as accessible with conventional DTI, were available.

In regions where *C*_c_ was found to be significantly different between the two groups, as for example in the parieto-occipital lobes and the dorsal parts of corpus callosum, changes to the microstructure could also be subject to additional interpretations including disruptions in tissue integrity in the form of loss of fibre coherence. In the neuropsychological evaluation,^7^ patients with white matter lesions in the brain MRI had a lower visuospatial index compared to those with normal MRI findings. The parietal lobes are important in the integration of sensory input, and the localized finding of changes in orientational coherence (*C*_c_) in the dorsal part of the brain could be related to affected visuospatial performance.

The variance in compartments’ size (*C*_MD_) was among the metrics showing widespread change between the control and patient groups. *C*_MD_ has previously been connected to cell density,^29^ where higher *C*_MD_ values stood for low cell density, and vice versa. Thus, the increase in *C*_MD_ observed in this cohort may be indicative of white matter damage in the form of cellular membrane disruption, cell swelling, cellular atrophy and necrosis.

Age-related effects on the considered QTI-derived metrics were also investigated. Consistent with other studies,^32,56^ we observed a decrease in FA and µFA and an increase in MD with increasing age. *C*_c_ was also found to decrease with age while *C*_MD_ seems to not be affected by aging.

The Fazekas scores (see Table 1) are slightly higher in the patient group, but no study participant had a Grade 3, meaning that the white matter changes are generally minimal to moderate. The white matter hyperintensities reflect damaged white matter, which is in line with the findings of the diffusion analysis. Studies have shown that white matter lesions are common in the acute/subacute phase of the disease but also persist at follow-up after COVID-19.^5,6,10^ The white matter thus seems to be affected on the micro- as well as on the macrolevel.

### Limitations

Several possible limitations need mentioning with respect to the adopted methodology for the data analysis. First, the accuracy and precision of the microstructural metrics as obtained via QTI were recently investigated.^59^ It was found that when such metrics are retrieved via QTI, they tend to be inaccurate in voxels presenting large variations in compartment size and/or high degree of orientation dispersion. While this should not severely affect the analysis of healthy white matter, care should be taken when considering, for example, oedematous fibrous tissue. Moreover, it was also recently reported that QTI metrics could be severely biased in noisy^41,59^ and under-sampled^41^ data. This issue was however recently addressed,^40,41^ and the estimation framework employed in this work should produce robust estimates with respect to these two issues.

Second, the adopted method assumes no diffusion time dependence; thus, contributions of restriction and exchange on the diffusion signal are not captured.^60^ Recent studies^61-63^ have shown that such contributions, while subtle, may not be negligible in the human brain; thus, the metrics considered here may be biased by neglecting them. Therefore, in future studies, methods including restriction effects and time dependence^64-66^ and exchange^67^ should be considered. Note that similar limitations apply to other studies employing DTI and NODDI.

Third, while TBSS is currently the most adopted method for comparing groups based on white matter diffusion metrics, concerns regarding the reliability of the different steps included in the framework have been raised.^68,69^ In particular, the results may depend on the selected target for the registration of all subjects to a common template, the performance of the registration algorithm and on the user’s choices for different settings.^68^ In this study, we adhered to the default and recommended settings as stated in the TBSS documentation (https://fsl.fmrib.ox.ac.uk/fsl/fslwiki/TBSS/UserGuide) since this should provide ground for comparison with other studies.^68^ Moreover, while the results shown here were obtained by using the FMRIB58_FA template as target for the registration, we also repeated the analysis using one of the healthy controls’ FA as template to check for results’ dependency on this step. Indeed, the results do not match perfectly, since the derived skeletons differ. However, they showed the same trends with respect to direction of change for the different QTI-derived metrics and their localization in the brain. Nevertheless, for this and other studies employing the TBSS framework, we advocate for caution when interpreting the results for specific tracts or anatomical structures.

Additional limiting factors for the interpretation of the results obtained in this study arise from the rather small cohort and the cross-sectional design which omits the longitudinal perspective on the development of brain-associated changes after COVID-19. The infectious status of the control group was not investigated, which might be considered a limitation. However, the inclusion criterion for the patient group was a previous hospitalization for COVID-19 with persisting symptoms at follow-up. Hence, the controls were recruited and included regardless of their previous infectious state if they were in good health with no symptoms of a post-COVID condition nor a previous hospitalization due to COVID-19. The differences found between the two groups might therefore be contributed to the course of the disease, where the hospitalization for COVID with persisting symptoms at follow-up is reflected in the deviating findings of the diffusion analysis.

The use of sex as a covariate in the statistical analysis may also be questioned since one group consisted of males only. We therefore repeated the analysis both not including the sex covariate and excluding the female subjects. When not using sex as a covariate in the group analysis, two of the statistical maps (AD and *C*_MD_) change. For *C*_MD_, the percentage of significant voxels increases by 15% if a sex covariate is included, whereas for AD, 90% of the otherwise significant voxels become non-significant when a sex covariate is used. Furthermore, repeating the group analyses with only male subjects (11 controls and 16 patients) resulted in statistical maps that are essentially unchanged to those where sex is used as a covariate (when analysing all 32 subjects; see Supplementary material). Given these results, we believe that including a sex covariate provides the most sound results.

Hospitalization and ventilator care might also have contributed to the observed changes, which can therefore not be attributed solely to the disease. Additionally, to the presented analysis, we performed permutation tests to assess whether the length of stay in ventilation care had any effect on the microstructure. Given that for this analysis we considered only the 16 subjects in the patient group (11 of which required ventilation care), no statistically significant results emerged. However, the trends were in agreement with the results obtained from the TBSS analysis: decreasing FA, µFA and *C*_c_ and increasing MD, AD, RD and *C*_MD_, with increasing length of time on ventilator. As length of time on ventilator is expected to correlate with the severity of the COVID-19, these results are not surprising and suggest that the infection indeed plays a role in the detected alterations to the brain white matter microstructure.

Ultimately, histopathology would be needed for a complete characterization of the observed tissue changes^70,71^; nevertheless, dMRI still provides a safe and non-invasive means for assessing the brain microstructure.

## Conclusion

In this cohort of patients who suffered from COVID-19 requiring hospitalization and with persisting symptoms at follow-up, we find general changes affecting the microstructure of the white matter of the brain, detectable with advanced dMRI. In particular, the QTI metrics *C*_MD_ and µFA demonstrated higher sensitivity to these alterations compared to the DTI metrics FA and MD. The observed changes, which are consistent with axonal damage, demyelination and oedema, might be a contributing factor to the diversity of central nervous system symptoms that many patients experience after COVID-19.

## Supplementary Material

fcad284_Supplementary_Data

## Data Availability

Due to the ethical permit and patient secrecy legislation, the images cannot be shared. Group results from the TBSS analysis are available at NeuroVault (https://identifiers.org/neurovault.collection:13799).
