# Supplementary material for: MRI with generalized diffusion encoding reveals damaged white matter in patients previously hospitalized for COVID-19 and with persisting symptoms at follow-up
Source: Brain Commun. 2023 Oct 22;5(6):fcad284. doi: 10.1093/braincomms/fcad284 (PMC10638510; doi:10.1093/braincomms/fcad284)
Supplement: fcad284_Supplementary_Data [file fcad284_supplementary_data.pdf]

Supplementary material

Here we show the comparison between the statistical analysis results presented in the main manuscript, and the repeated analyses where either the female subjects were excluded, or sex was not included as a covariate in the model.

For each microstructure metric, the first row (I) shows the results of the statistical analysis performed on the 32 subjects, with age and sex as covariates. This corresponds to the results presented in the manuscript. The second row (II) shows the results obtained by excluding the female subjects, thus comparing 11 controls with 16 patients. The third row (III) shows the results obtained considering 32 subjects, but including only age as a covariate in the model.

As reported in the discussion section, the results in rows I and II are essentially unchanged. Conversely, the results in rows I and III present the most prominent differences in the AD and CMD maps, while the results for the other considered metrics were affected to a much lesser extent. However, not including sex as a covariate generally results in more voxels presenting statistically significant differences between the two groups, and smaller p-values.

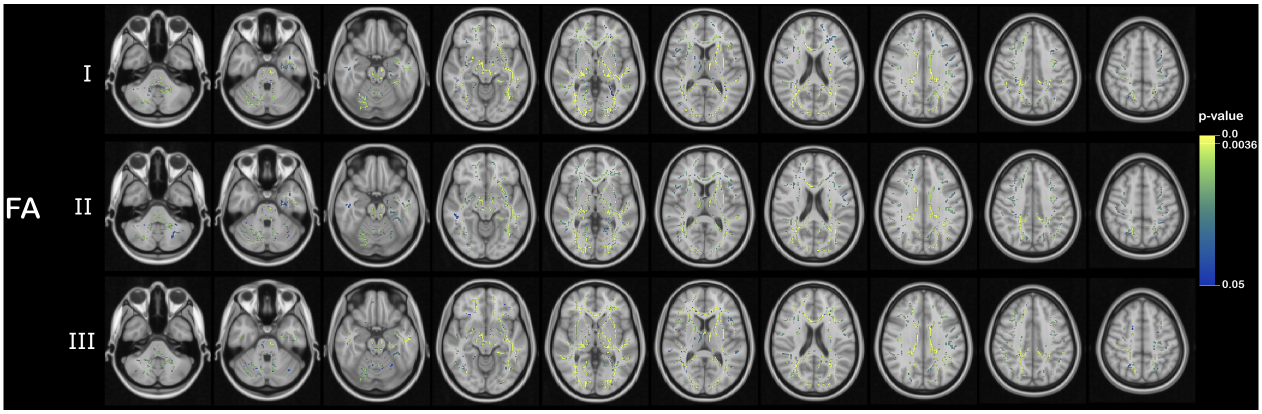

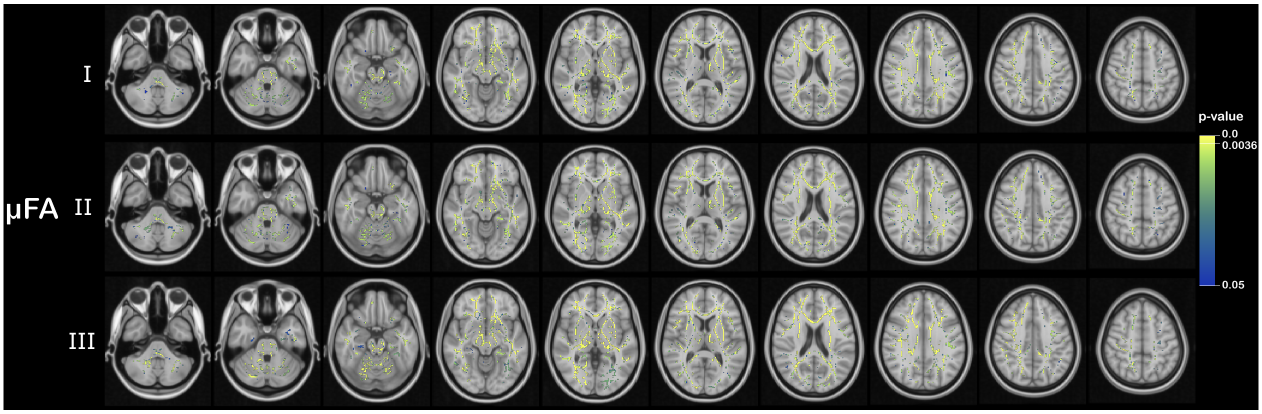

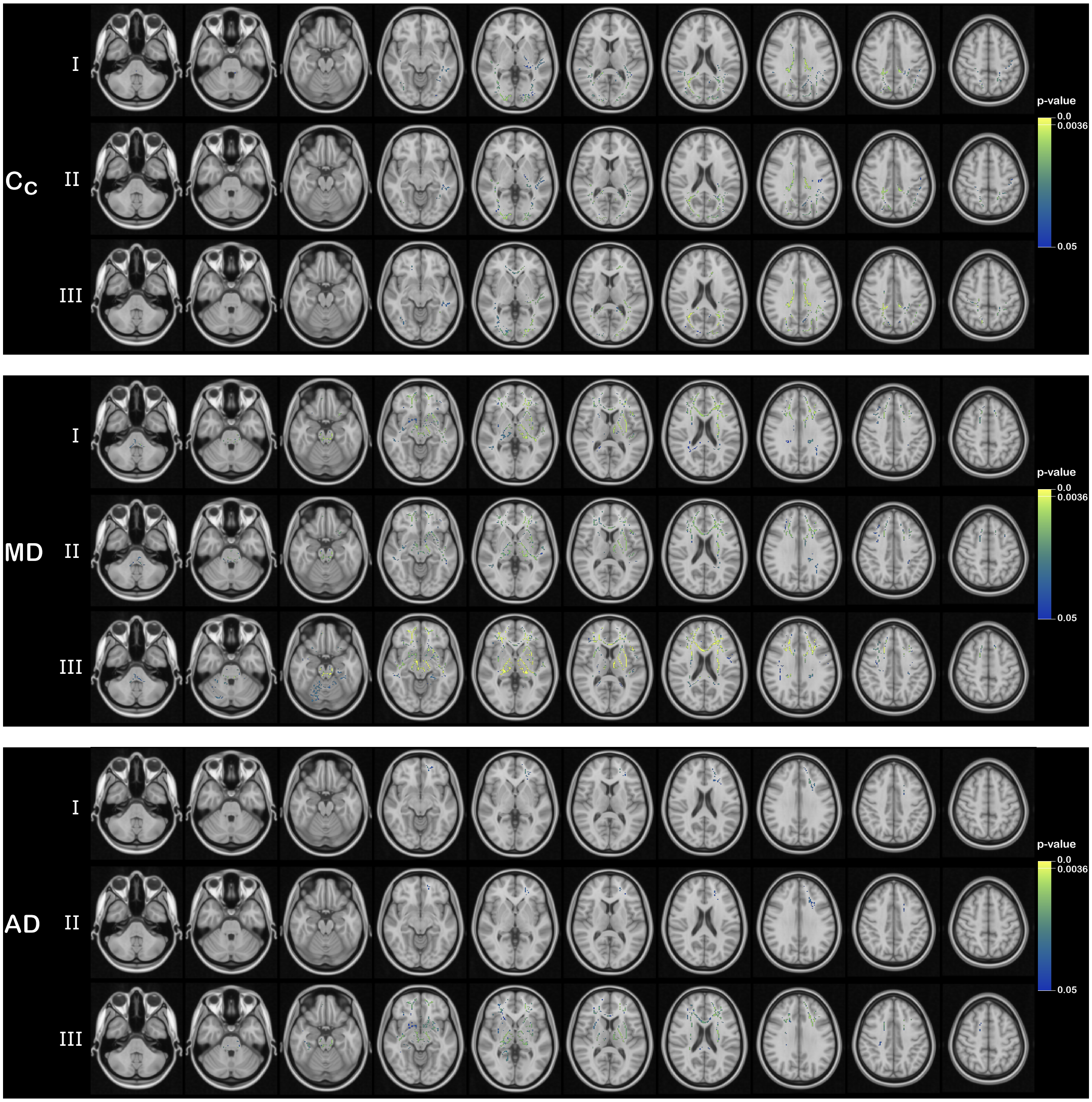

CC
MD
AD
I
II
III
p-value
0.0
0.0036
0.05

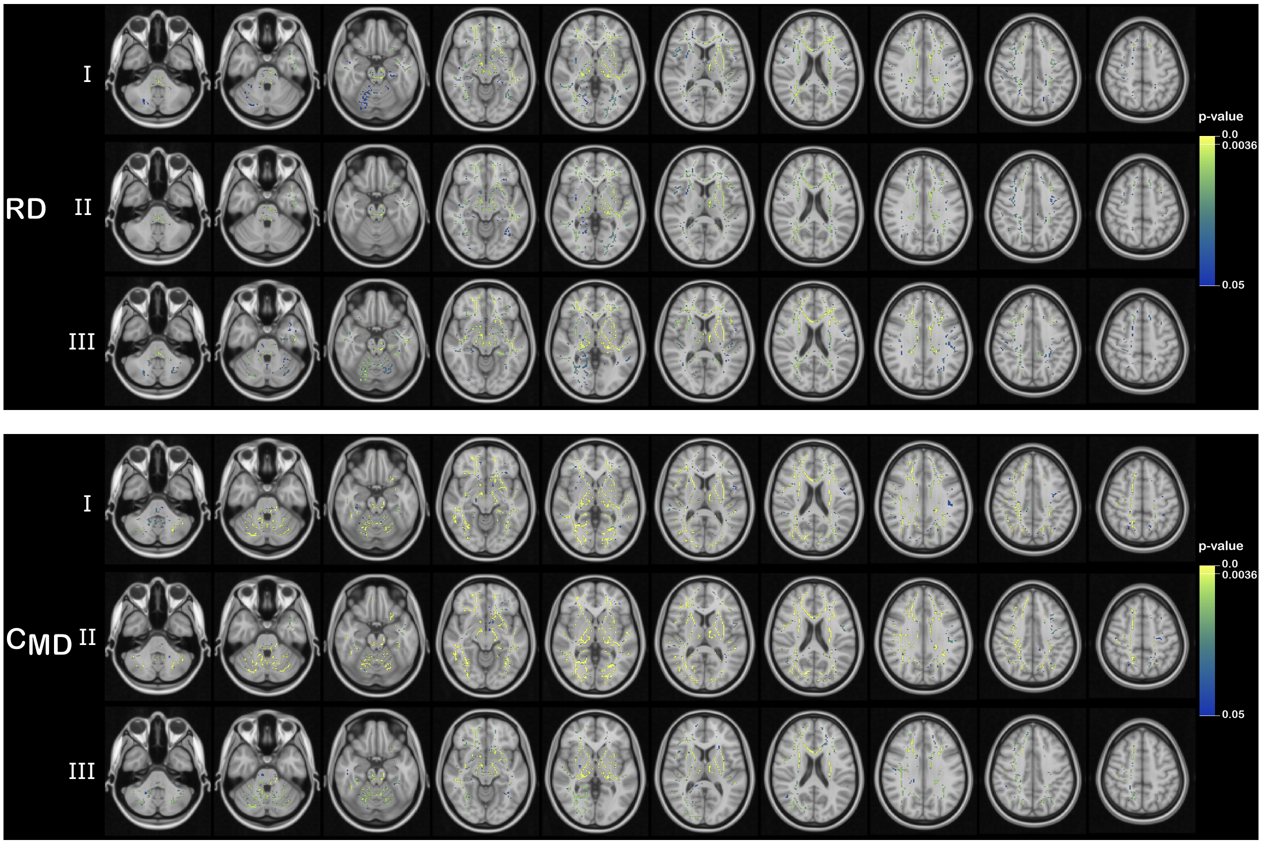

RD
I
II
III
p-value
0.0
0.0036
0.05
CMD
I
II
III
p-value
0.0
0.0036
0.05
